# Supplementary material for: Transcriptomics and proteomics reveal a cooperation between interferon and T-helper 17 cells in neuromyelitis optica
Source: Nat Commun. 2020 Jun 5;11:2856. doi: 10.1038/s41467-020-16625-7 (PMC7275086; doi:10.1038/s41467-020-16625-7)
Supplement: Supplementary file 1 — Supplementary Information [file 41467_2020_16625_MOESM1_ESM.pdf]

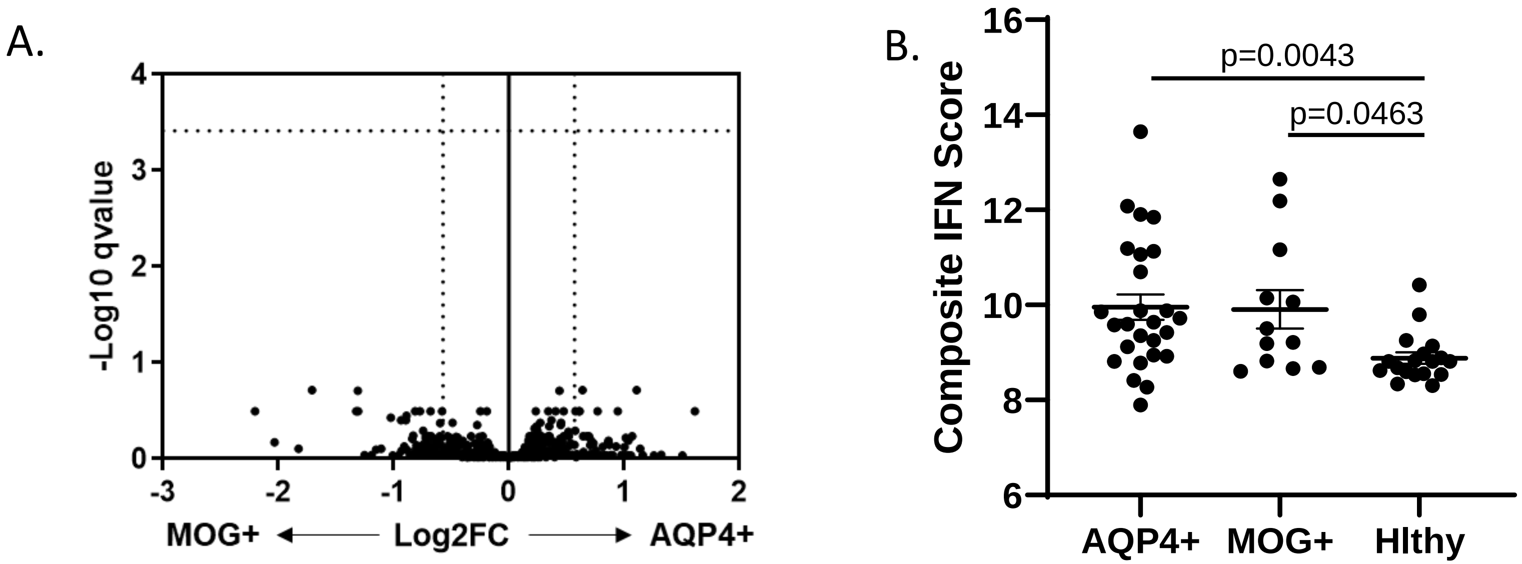

Supplemental Figure 1: A. Comparison of RNA profiles of MOG+ and AQP4+ NMOSD patients. Dashed line represents the 0.05 - q-value and 0.67 Log 2FC. **B.** Composite IFN scores (average read count of IFN-I genes) of AQP4-Ig+ and MOG-Ig+ NMOSD patients. P-values were determined using two-tailed Anova tests with multiple comparisons corrected by the Dunnett’s method. Error bars indicate the S.E.M.

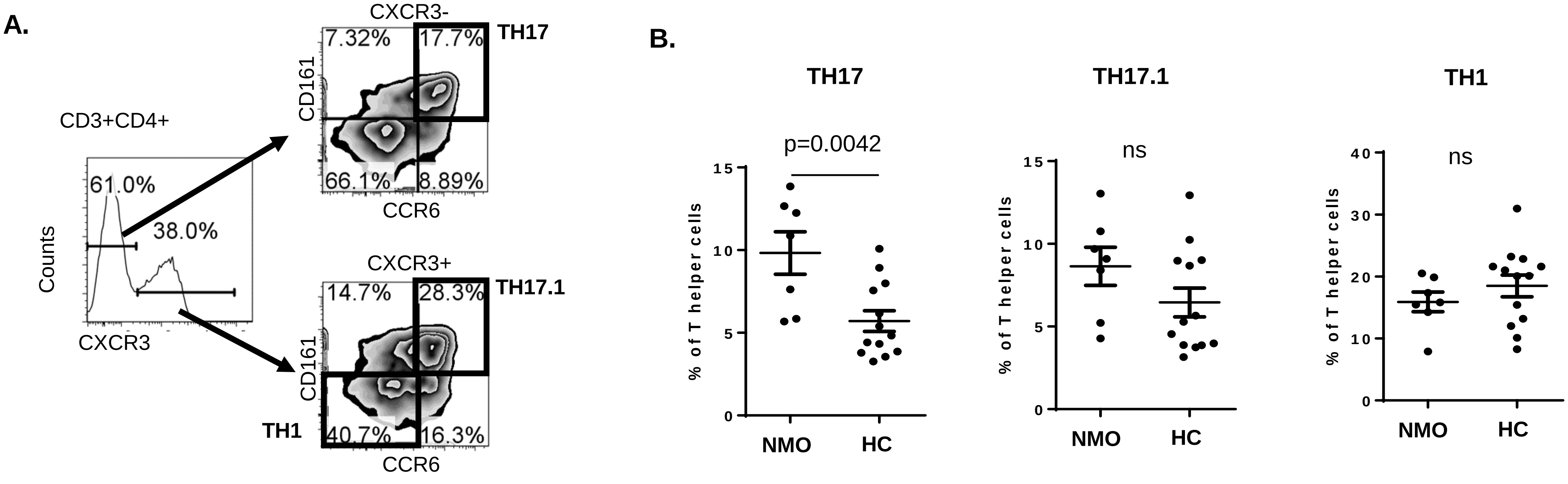

Supplemental Figure 2: Comparison of T cell subsets in PBMCs of NMOSD patients (n=7) versus Healthy Controls (n=13). (A) Gating strategy of TH1, TH17 and TH17.1 cell populations based on the expression of CD3, CD4, CXCR3, CCR6 and CD161. (B) Percentage of TH17 cells (CXCR3-CCR6+CD161+), TH17.1 cells (CXCR3-CCR6+CD161+) and TH1 cells in NMOSD patients compared to healthy controls (HC). Two-tailed Mann Whitney tests were used to determine significance. Statistical Error bars indicate the S.E.M.

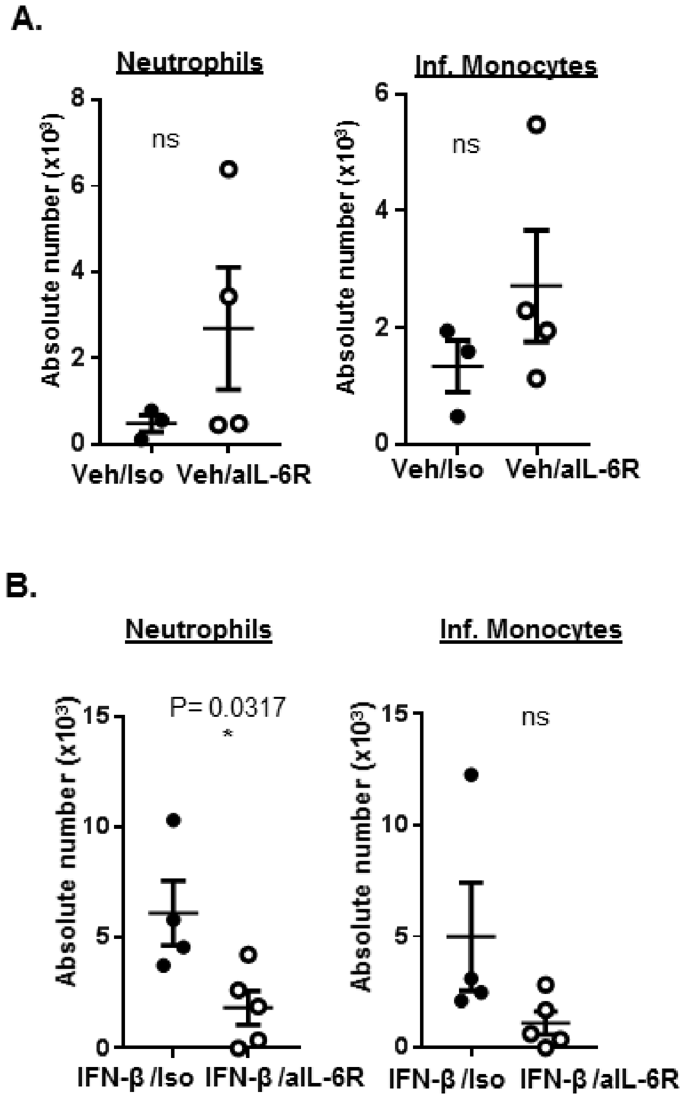

Supplemental Figure 3: Effects of IL-6 blockade on the infiltration of macrophages and neutrophils. TH17-EAE mice, either treated with IFN-β or vehicle, were also treated with either anti-IL-6R or an isotype control every 5 days from days 1-11. (A) Number of neutrophils and inflammatory monocytes in spinal cords of vehicle-treated mice (day 37). (B) Number of neutrophils and inflammatory monocytes in spinal cords of IFN-β-treated EAE mice (day 37). Statistical analysis was performed using two-tailed Mann Whitney tests. ($P < 0.05$ were considered significant*). Error bars indicate the S.E.M.

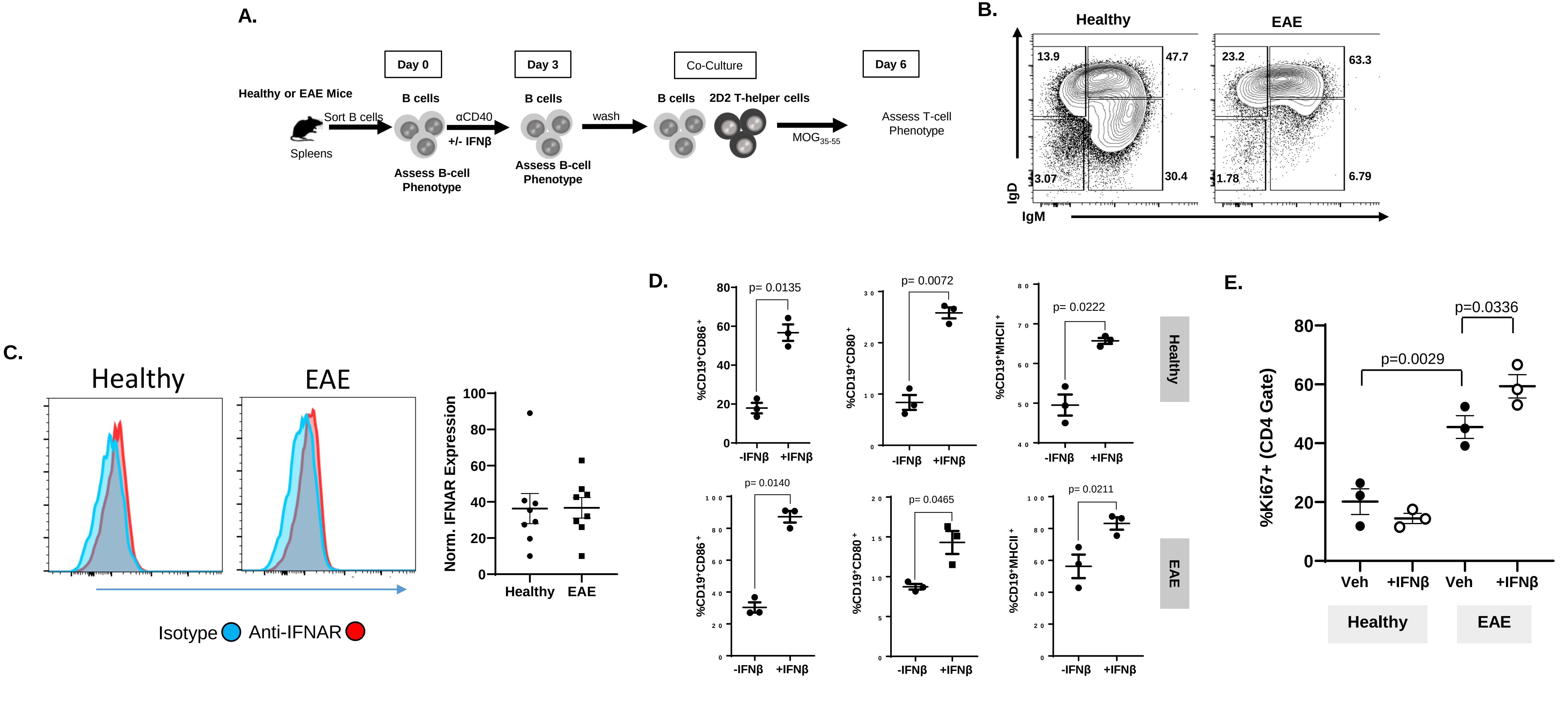

Supplemental Figure 4. (**A**) Schematic diagram illustrating that purified B cells from spleens of healthy or EAE mice (*n* = 3-5 per group) were stimulated with anti-CD40 ± IFN-β for 3 days. Following stimulation, B cells were washed and co-cultured with $CD4^+$ T cells from 2D2 mice in the presence of $MOG_{35\text{-}55}$ antigen for 3 more days. Prior to stimulation with IFN-β, B cell phenotype was determined for **(B)** IgD, IgM and **(C)** IFNAR expression. (**D**) B cells from healthy and EAE mice (*n=3* per group) were stimulated with αCD40 ± IFN-β. Following stimulation, expression of CD80, CD86 and MHCII by B cells was assessed by flow cytometry. Statistical significance was determined using two tailed Student t-tests. **(E)** Stimulated B cells were washed and co-cultured with antigen-specific 2D2 T-helper cells in the presence of $MOG_{35\text{-}55}$ antigen. Proliferation of the 2D2 $CD4^+$ T cells was measured by Ki-67 staining by flow cytometry. A two-tailed paired Anova test with multiple comparison corrected by the Holm-Sidak's method was used to determine statistical significance. P values < 0.05 were considered significant. Error bars indicate s.e.m.

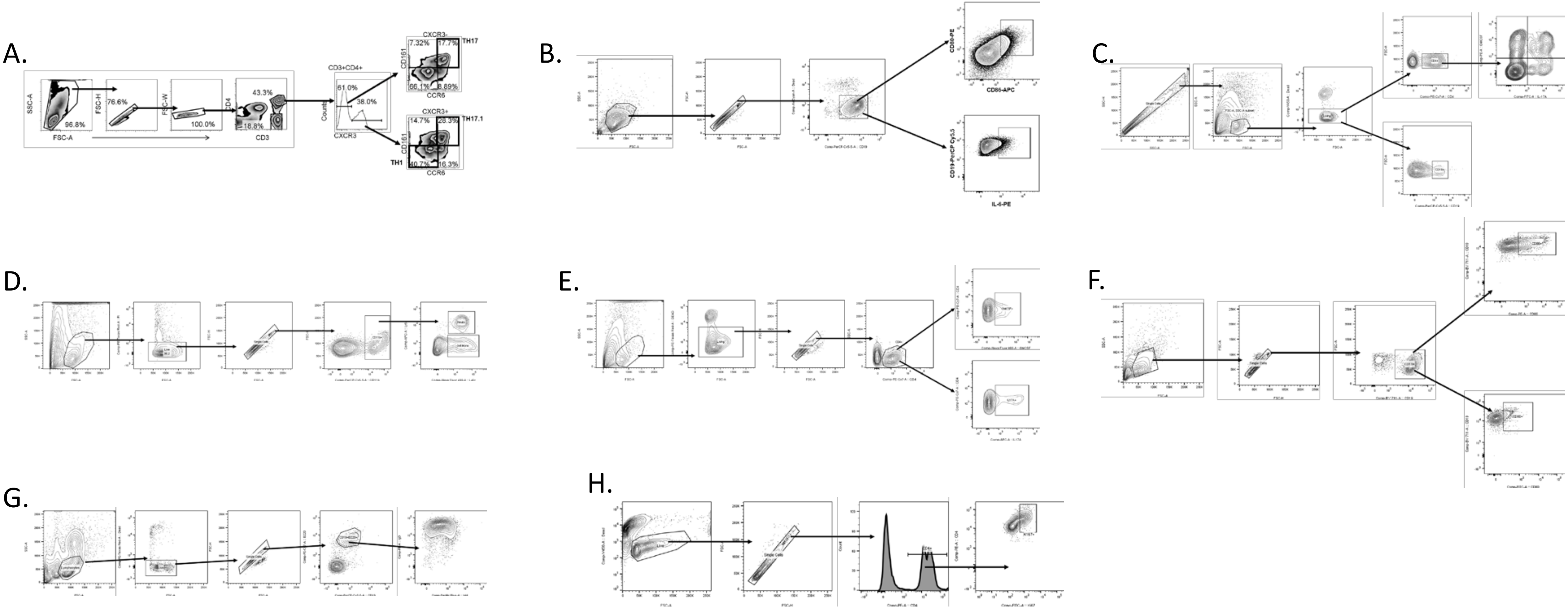

Supplemental Figure 5: Flow cytometric gating strategies for A) figure 1m-p and supplemental figure 2, B) figure 3b-c, C) figure 4d, e and f, D) supplemental figure 3, E) figure 5b, d, f and h, F) figure 6a, b and supplemental figure 4d, G) supplemental figure 4 b and c, H) figure 6d, e and supplemental figure 4e.

Supplemental Table 1. NMOSD patient clinical and demographic information.

Variables	NMOSD
Total no.	42
Age, year, mean (SD)	48.2 (13.6)
Sex F/M (%F)	36/6 (85.7)
Race/Ethnicity, N (%)	
African	1 (2.4)
Arab	2 (4.8)
Asian	1 (2.4)
Caucasian	37 (88.0)
unknown	1 (2.4)
Serological status, N (%)	
AQP4-Ig+	30 (71.4)
MOG-Ig+	12 (28.6)
Treatment, N (%)	
Rituximab/Ocreluzumab	26 (61.9)
none	7 (16.6)
Azathioprine	5 (11.8)
Mycophenolate	2 (4.8)
Belimumab	1 (2.4)
unknown	1 (2.4)
EDSS, mean (range)	3.14 (0 - 6.5)

Notes:
EDSS is Expanded Disability Status Scale.

Supplemental Table 2. Michigan NMOSD patient cohort: Clinical & demographic information.

**Variables**	**NMOSD**
Total no.	6
Age, year, mean (SD)	45.6 (17.3)
Sex F/M (%F)	4/2 (67.7)
Serological status, N (%)	
AQP4-Ig+	6 (100)
MOG-Ig+	0 (0)
Treatment, N (%)	
None	6 (100)
EDSS, mean (range)	5.2 (3 - 6.5)

Notes:
EDSS is Expanded Disability Status Scale.
