# Supplementary material for: Transcriptomics and proteomics reveal a cooperation between interferon and T-helper 17 cells in neuromyelitis optica
Source: Nat Commun. 2020 Jun 5;11:2856. doi: 10.1038/s41467-020-16625-7 (PMC7275086; doi:10.1038/s41467-020-16625-7)
Supplement: Supplementary file 3 — Reporting Summary [file 41467_2020_16625_MOESM3_ESM.pdf]

nature research

Corresponding author(s): Robert Axtell

Last updated by author(s): Apr 29, 2020

Reporting Summary

Nature Research wishes to improve the reproducibility of the work that we publish. This form provides structure for consistency and transparency in reporting. For further information on Nature Research policies, see Authors & Referees and the Editorial Policy Checklist.

Statistics

For all statistical analyses, confirm that the following items are present in the figure legend, table legend, main text, or Methods section.

n/a	Confirmed	
☐	☒	The exact sample size (n) for each experimental group/condition, given as a discrete number and unit of measurement
☐	☒	A statement on whether measurements were taken from distinct samples or whether the same sample was measured repeatedly
☐	☒	The statistical test(s) used AND whether they are one- or two-sided<br>*Only common tests should be described solely by name; describe more complex techniques in the Methods section.*
☐	☒	A description of all covariates tested
☐	☒	A description of any assumptions or corrections, such as tests of normality and adjustment for multiple comparisons
☐	☒	A full description of the statistical parameters including central tendency (e.g. means) or other basic estimates (e.g. regression coefficient) AND variation (e.g. standard deviation) or associated estimates of uncertainty (e.g. confidence intervals)
☐	☒	For null hypothesis testing, the test statistic (e.g. F, t, r) with confidence intervals, effect sizes, degrees of freedom and P value noted<br>*Give P values as exact values whenever suitable.*
☒	☐	For Bayesian analysis, information on the choice of priors and Markov chain Monte Carlo settings
☒	☐	For hierarchical and complex designs, identification of the appropriate level for tests and full reporting of outcomes
☐	☒	Estimates of effect sizes (e.g. Cohen's d, Pearson's r), indicating how they were calculated

Our web collection on statistics for biologists contains articles on many of the points above.

Software and code

Policy information about availability of computer code

Data collection	None
Data analysis	none

For manuscripts utilizing custom algorithms or software that are central to the research but not yet described in published literature, software must be made available to editors/reviewers. We strongly encourage code deposition in a community repository (e.g. GitHub). See the Nature Research guidelines for submitting code & software for further information.

Data

Policy information about availability of data

All manuscripts must include a data availability statement. This statement should provide the following information, where applicable:

- Accession codes, unique identifiers, or web links for publicly available datasets
- A list of figures that have associated raw data
- A description of any restrictions on data availability

All relevant data are available from the authors and the RNA-seq data has been deposited in NCBI/GEO

Field-specific reporting

Please select the one below that is the best fit for your research. If you are not sure, read the appropriate sections before making your selection.

☒ Life sciences ☐ Behavioural & social sciences ☐ Ecological, evolutionary & environmental sciences

For a reference copy of the document with all sections, see nature.com/documents/nr-reporting-summary-flat.pdf

Life sciences study design

All studies must disclose on these points even when the disclosure is negative.

Sample size	We used the following to determine sample size. Sample size estimates are provided for identifying fold changes (FC) of 1.75, 2.0, and 2.25 assuming 5% type 1 error, sequencing depth of 50 million mapped reads, and an estimate of the biological variation = 0.6 (Fig. 12). At 80% power, per group sample sizes of 9, 12, and 19 are able detect FC of 2.25, 2.0, and 1.75, respectively. At 90% power, these per group sample sizes increase to 12, 17, and 25. Based on our experience, a FC≥2 is required to limit the number of DE transcripts generated by RNA-seq. Because of the increased sensitivity of RNA-seq to detect DE transcripts, we found that in our whole blood experiment (preliminary data), any FC lower than 2 exponentially increased the number of transcripts, leaving us with an unmanageably massive number to evaluate. Of course, since we will be working with more homogeneous cell subsets in parts of this aim, we will re-evaluate this cut-off as we work with the data. These results demonstrate that we have more than sufficient power (essentially 100%) to detect significant differences in expression comparisons of 25 NMO cases and 25 controls. Power was calculated using the "Calculating Sample Size Estimates for RNA Sequencing Data" algorithm.
Data exclusions	no data were excluded.
Replication	For the RNA-Seq data of NMO patient samples, we have not replicated these data. NMO is a rare patient population and cohorts with well characterized clinical history with match serum, pax-gene tubes and PBMCs are not easily available. Many serum proteins identified in our study have been shown to be elevated NMOSD in previous studies.<br><br>For animal studies, we replicated these experiments at least 2-4 times with similar results with each experiment.
Randomization	Treatment of the EAE animals were randomized before the induction of disease. Mice were all age and sex matched in each treatment group.
Blinding	The personnel performing assays were blinded from the clinical and demographic data associated with the samples.

Reporting for specific materials, systems and methods

We require information from authors about some types of materials, experimental systems and methods used in many studies. Here, indicate whether each material, system or method listed is relevant to your study. If you are not sure if a list item applies to your research, read the appropriate section before selecting a response.

Materials & experimental systems

n/a	Involved in the study	
☐	☒	Antibodies
☒	☐	Eukaryotic cell lines
☒	☐	Palaeontology
☐	☒	Animals and other organisms
☐	☒	Human research participants
☒	☐	Clinical data

Methods

n/a	Involved in the study	
☒	☐	ChIP-seq
☐	☒	Flow cytometry
☒	☐	MRI-based neuroimaging

Antibodies

Antibodies used	Anti-Human antibodies:<br>1. Viability dye:<br>Supplier: Tonbo<br>Cat# 13-0870-T500<br>Clone name: N/A<br>Lot# D0870113016133<br><br>2. CD19<br>Supplier: Biolegend<br>Cat# 302230<br>Clone name: HIB19<br>Lot# B268527<br><br>3. CD86<br>Supplier: Biolegend<br>Cat# 305412<br>Clone name: IT2.2<br>Lot# B239207<br><br>4. CD86<br>Supplier: Biolegend<br>Cat# 305208

Clone name: 2D10
Lot# B214339
5. IL-6
Supplier: Biolegend
Cat# 501107
Clone name: MQ2-13A5
Lot# B235510

Anti-mouse antibodies:
1.Viability dye
Supplier: eBioscience
Cat# 65-0863-14
Clone name: N/A
Lot# 2143488

2. F4/80
Supplier: Biolegend
Cat# 123123
Clone name: BM8
Lot# B217178

3. Ly6C
Supplier: Biolegend
Cat# 128035
Clone name: HK1.4
Lot# B266314

4. Ly6G
Supplier: Biolegend
Cat# 127610
Clone name: 1A8
Lot# B204928

4. IgD
Supplier: Biolegend
Cat# 405506
Clone name: 11-26c.2a
Lot# --

4. CD19
Supplier: Biolegend
Cat# 115555
Clone name: 6D5
Lot# B298801

4. CD86
Supplier: Biolegend
Cat# 105008
Clone name: GL-1
Lot# B207219

4. IFNAR
Supplier: Biolegend
Cat# 127311
Clone name: MAR1-5A3
Lot# B251509

4. IgG1
Supplier: Biolegend
Cat# 400111
Clone name: MOPC-21
Lot# --

4. CD80
Supplier: Biolegend
Cat# 104721
Clone name: 16-10A1
Lot# B169842

4. CD11b
Supplier: eBioscience
Cat# 48-0112-82
Clone name: M1/70
Lot# E10253-1632

4. MHCII
Supplier: eBioscience
Cat# 25-5321-82
Clone name: M5/114.15.2
Lot# --

4. IgM
Supplier: eBioscience
Cat# 48-5790-82
Clone name: II/41
Lot# 4344367

4. CD4
Supplier: eBioscience
Cat# 25-0041-82
Clone name: GK1.5
Lot# 25-0041-82

4. CD4
Supplier: BDBioscience
Cat# 553730
Clone name: GK1.5
Lot# 4101767

4. IL-17
Supplier: Biolegend
Cat# 506908
Clone name: TC11-18H10.1
Lot# B167210

4. GM-CSF
Supplier: Biolegend
Cat# 505406
Clone name: MP1-22E9
Lot# B196270

4. Ki67
Supplier: Biolegend
Cat# 151204
Clone name: 11F6
Lot# B275639

Validation

All antibodies are from commercially available and have been used extensively. Below are links to these products.
Link: https://tonbobio.com/products/ghost-dye-violet-510
Link: https://www.biolegend.com/en-us/products/percp-cyanine5-5-anti-human-cd19-antibody-4226
Link: https://www.biolegend.com/en-us/products/apc-anti-human-cd86-antibody-2864
Link: https://www.biolegend.com/en-us/products/pe-anti-human-cd80-antibody-554
Link: https://www.biolegend.com/en-us/products/pe-anti-human-il-6-antibody-982
Link: https://www.thermofisher.com/order/catalog/product/65-0863-14?SID=srch-hj-65-0863-14#/65-0863-14?SID=srch-hj-65-0863-14
Link: https://www.biolegend.com/en-us/products/pacific-blue-anti-mouse-f4-80-antibody-4075
Link: https://www.biolegend.com/en-us/products/brilliant-violet-605-anti-mouse-ly-6c-antibody-8727
Link: https://www.biolegend.com/en-us/products/alexa-fluor-647-anti-mouse-ly-6g-antibody-4780
Link: https://www.biolegend.com/en-us/products/pe-anti-mouse-igd-1379
Link: https://www.biolegend.com/en-us/products/brilliant-violet-711-anti-mouse-cd19-antibody-12075
Link: https://www.biolegend.com/en-us/products/pe-anti-mouse-cd86-antibody-256
Link: https://www.biolegend.com/en-us/products/pe-anti-mouse-ifnar-1-antibody-4784
Link: https://www.biolegend.com/en-us/products/pe-mouse-igg1--kappa-isotype-ctrl-1408
Link: https://www.biolegend.com/en-us/products/percpcyanine55-anti-mouse-cd80-antibody-4275
Link: https://www.thermofisher.com/antibody/product/CD11b-Antibody-clone-M1-70-Monoclonal/48-0112-82
Link: https://www.thermofisher.com/antibody/product/MHC-Class-II-I-A-I-E-Antibody-clone-M5-114-15-2-Monoclonal/25-5321-82
Link: https://www.thermofisher.com/antibody/product/IgM-Antibody-clone-II-41-Monoclonal/48-5790-82
Link: https://www.thermofisher.com/antibody/product/CD4-Antibody-clone-GK1-5-Monoclonal/25-0041-82
Link: https://www.bdbiosciences.com/us/applications/research/t-cell-immunology/th-1-cells/surface-markers/mouse/pe-rat-anti-mouse-cd4-gk15/p/553730
Link: https://www.biolegend.com/fr-fr/products/fitc-anti-mouse-il-17a-antibody-3534
Link: https://www.biolegend.com/en-us/products/pe-anti-mouse-gm-csf-antibody-958
Link: https://www.biolegend.com/en-us/products/alexa-fluor-488-anti-mouse-human-ki-67-antibody-12889

Animals and other organisms

Policy information about studies involving animals; ARRIVE guidelines recommended for reporting animal research

Laboratory animals	8-12 week old Female mice in the C57BL/6 background were used in this study.
Wild animals	none
Field-collected samples	none
Ethics oversight	The OMRF IACUC approved all procedures used in this manuscript.

Note that full information on the approval of the study protocol must also be provided in the manuscript.

Human research participants

Policy information about studies involving human research participants

Population characteristics	Diagnosis: Neuromyelitis Optica or Healthy. Age range: 22-70 years. Sex; 85% female.
Recruitment	Written informed consent was obtained from individuals prior to participation in the study.
Ethics oversight	This study was approved by the Charité Universitätsmedizin Berlin, University of Michigan and the Oklahoma Medical Research Foundation's Institutional Review Boards.

Note that full information on the approval of the study protocol must also be provided in the manuscript.

Flow Cytometry

Plots

Confirm that:

☒ The axis labels state the marker and fluorochrome used (e.g. CD4-FITC).

☒ The axis scales are clearly visible. Include numbers along axes only for bottom left plot of group (a 'group' is an analysis of identical markers).

☒ All plots are contour plots with outliers or pseudocolor plots.

☒ A numerical value for number of cells or percentage (with statistics) is provided.

Methodology

Sample preparation	Human PBMCs: Heparinized blood was collected from each patient in BD VacutainerTM Sodium HeparinN green top tubes. The green top tubes were mixed and centrifuged for isolation of PBMCs at the cell layer.<br>Mouse immune cells: CNS infiltration by immune cells was assessed by perfusing EAE mice with PBS and collecting their brains and spinal cords. CNS tissue was homogenized through mechanical disruption and homogenates were incubated with DNAse (5 µl/ml; Sigma) and collagenase (4 mg/ml; Roche) at 37 °C for 1 hour. Cells were isolated using a Percoll gradient and analyzed by FACS.
Instrument	BD FACSCantoII or BD LSR2
Software	FlowJo 10.6.1
Cell population abundance	Not applicable. We did not Sort cells.
Gating strategy	All cells were stained with a fixable viability dye. Gating was on the Viability Dye negative cell population. Viable cells would then be gated on single cells defined by the diagonal FSC-H/FSC-A. Single cells would then be gated on FSC SCC to assess immune cell subsets. Surface Markers used (CD4, CD19, CD11b, Ly6C, Ly6G, MHC) have clear positive and negative cell populations. Positive and negative intracellular cytokines and Ki67 were based on unstimulated cells.

☒ Tick this box to confirm that a figure exemplifying the gating strategy is provided in the Supplementary Information.
