# Supplementary material for: Transcriptomics and proteomics reveal a cooperation between interferon and T-helper 17 cells in neuromyelitis optica
Source: Nat Commun. 2020 Jun 5;11:2856. doi: 10.1038/s41467-020-16625-7 (PMC7275086; doi:10.1038/s41467-020-16625-7)
Supplement: Supplementary file 4 — Description of Additional Supplementary Files [file 41467_2020_16625_MOESM4_ESM.pdf]

Description of Additional Supplementary Files

Supplementary Data 1. Genes expressed with q values less than 0.05 when comparing NMOSD on Rituximab therapy vs Healthy controls. A Wald test was used for calculating p value and Benjamini-Hochberg procedure was used for q value (FDR) calculation.

Supplementary Data 2. Genes expressed with q values less than 0.05 when comparing NMOSD on Other therapies vs Healthy controls. A Wald test was used for calculating p value and Benjamini-Hochberg procedure was used for q value (FDR) calculation.

Supplementary Data 3. Genes expressed with q values less than 0.05 when comparing Untreated NMOSD vs Healthy controls. A Wald test was used for calculating p value and Benjamini-Hochberg procedure was used for q value (FDR) calculation.

Supplementary Data 4. Protein analytes with p values less than 0.05 when comparing IFN-high and IFN-low NMOSD patients to Healthy controls. A two-way ANOVA with Dunnett's multiple comparisons test was used to determine significance.

Supplementary Data 5. Genes expressed with q values less than 0.05 when comparing NMOSD patients on Rituximab therapy vs patients not on Rituximab therapy. Genes are grouped according to the respective gene cluster. A Wald test was used for calculating p value and Benjamini-Hochberg procedure was used for q value (FDR) calculation.

Supplementary Data 6. Protein analytes with p values less than 0.05 when comparing IFN-high and IFN-low NMOSD patients in B cell-deficient and B cell-sufficient groups vs Healthy controls. A two-way ANOVA with Dunnett's multiple comparisons test was used to determine significance.
